# Editorial: Regulators of the immune-tumor microenvironment: a new frontier for cancer immunotherapy

**DOI:** 10.3389/fmed.2026.1764720

**Published:** 2026-02-09

**Authors:** Reem A. Assal, Rana A. Youness, Mai F. Ragab, Noha M. Elemam

**Affiliations:** 1 Department of Pharmacology and Toxicology, Faculty of Pharmacy, Heliopolis University for Sustainable Development (HU), Cairo, Egypt; 2 Department of Molecular Biology and Biochemistry, Molecular Genetics Research Team (MGRT), Faculty of Biotechnology, German International University (GIU), Cairo, Egypt; 3 School of Life and Medical Sciences, University of Hertfordshire Hosted by Global Academic Foundation, New Administrative Capital, Cairo, Egypt; 4 Department of Clinical Sciences, College of Medicine, University of Sharjah, Sharjah, United Arab Emirates; 5 Research Institute for Medical and Health Sciences, University of Sharjah, Sharjah, United Arab Emirates

**Keywords:** epigenetics, immune therapeutics, immune-oncology, solid malignancies, tumor microenvironment (TME)

The tumor microenvironment (TME) is a complex ecosystem whose immunosuppressive nature often causes resistance to cancer immunotherapy in solid malignancies. This editorial features the articles published in this Research Topic “*Regulators of the Immune-Tumor Microenvironment: A New Frontier for Cancer Immunotherapy*” which is published in Frontiers in Medicine. The introduced Research Topic focused on investigating the regulation of the immune-TME and identifying actionable epigenetic pathways to overcome the resistance to cancer immunotherapy in solid malignancies. The overarching goal is to unravel critical crosstalk mechanisms to develop novel, more effective therapies and improve patient outcomes. Also, this topic aimed to provide insights into various crosstalk mechanisms that could be translated into clinical practice.

Recent cancer research recognizes the critical role of the TME crosstalk, shifting focus to cancer-associated fibroblasts (CAFs). These cells, previously underestimated, are now considered essential for fostering immunosuppression and promoting malignant tumor growth. The review by Mukherjee et al. examines the origin and altered programs of CAFs, detailing their complex interplay with TME immune cells in solid tumors and B cell lymphomas. The article discusses CAFs' “reprogrammable” roles in immuno-regulation (suppression, activation, and avoidance) and their contribution to drug resistance. Finally, it explores therapeutic strategies designed to target CAFs as a means to enhance overall treatment response.

The focus on the TME's role in cancer progression extends beyond established biological factors to potentially modifiable systemic conditions. Lung cancer remains highly lethal, necessitating the identification of novel, reversible risk factors. Obstructive sleep apnea (OSA) is a common, often underestimated sleep disorder with unclear links to lung cancer. A potential biological relationship suggests the associated pathophysiological processes of OSA—like intermittent hypoxia and sleep fragmentation—may impair immune function and neuroendocrine regulation. This could contribute to lung cancer onset, accelerated progression, and treatment resistance. OSA may be a preventable contributor to disease progression, but its independent effect from other risk factors needs clarification. This review integrated literature on OSA and lung cancer to foster interdisciplinary research. Another study by Berzaghi et al. investigated a different aspect of stromal manipulation, examining how radiotherapy affects the immunomodulatory role of CAFs in the TME. Using human non-small cell lung cancer (NSCLC) CAFs, researchers applied single-high or fractionated radiation doses. Results showed that CAFs survived the radiation but entered senescence, yet they did not contribute to anti-tumor immune responses via DAMPs or IFN-I release. Crucially, the secretion of most immunoregulatory cytokines remained unchanged. However, radiation significantly upregulated key immunosuppressive receptors like CD73 and CD276 on the CAF surface. These findings suggest that irradiated CAFs may enhance the immunosuppressive state of the TME post-radiotherapy. Shifting the focus from stromal cells to soluble signaling molecules that govern the TME, the review by Jung and Paust examined the impact of chemokines on tumor immunity and metastasis and highlighted their potential in immunotherapy, particularly in lung cancer. Lung cancer is a prevalent malignancy with low survival rates and limited treatment options. Like many other solid tumors, its TME is a complex network that can hinder cancer immunotherapy due to its immunosuppressive nature. Chemokines play a vital role in immune cell signaling and migration within the TME, influencing tumor progression or suppression depending on the context. Moreover, this review explored recent chemokine-targeted research advancements in the cancer immunotherapy field.

The mini-review by Sami and Raza reported that breast cancer incidence and mortality are expected to rise significantly by 2040, driven by clonal evolution causing drug resistance. The TME creates an immunosuppressive niche, facilitating immune evasion. The review focused on tumor-associated macrophages (TAMs), which are critical TME components. Macrophages switch from an M1 (anti-tumor) to an M2 (pro-tumor) phenotype, prompted by cancer cells, thus mediating immune evasion. This review discussed the crucial role of TAMs for developing novel immunotherapies, such as engineered macrophages or M2-targeting drugs, to improve long-term patient survival. Aggressive cancers like triple-negative breast cancer require novel therapies. The study by Demeule et al. introduced Sudocetaxel Zendusortide, a peptide-drug conjugate that activates the cyclic GMP-AMP synthase/stimulator of interferon genes pathway. Findings show superior tumor inhibition vs. docetaxel and increased immune cell infiltration. The observed synergy with anti-programmed death-ligand 1 strongly supports its clinical development as an immuno-chemotherapeutic agent with checkpoint inhibitors.

Two studies investigated the role TME in HCC. Du et al. examined the immune escape in hepatocellular carcinoma (HCC), which is the sixth most common global cancer with high mortality rates. Tumor growth relies significantly on the immune system, with HCC achieving immune escape through the TME. This article reviews the function of regulatory T cells (Tregs) within the TME, emphasizing how Tregs inhibit and regulate various immune cells, cytokines, ligands, and receptors to promote this escape. Furthermore, it discusses the mechanism of CAR-T therapy for HCC and the critical relationship between CAR-T cells and Tregs. Furthermore, the review by Lu et al. aimed to consolidate current knowledge on the role of alpha-fetoprotein (AFP) in the TME of HCC. While AFP is a fetal protein, its elevated serum levels correlate significantly with HCC onset and progression in adults. Research highlights the TME as crucial for the malignant transformation of HCC, with AFP acting as a key promoter within the TME. The review analyzed the effects of AFP on various TME cells, its contribution to tumor immune evasion, and its clinical application in HCC diagnosis and treatment, offering valuable insights for improving clinical outcomes.

Ovarian cancer is a highly lethal gynecological malignancy, making its TME a key therapeutic target. Transglutaminase 2 (TG2) promotes tumor progression, but its role in the TME was unclear. This study, which employed novel irreversible TG2 inhibitors, revealed that they reduced invasiveness and extended survival in xenograft models. Using syngeneic mouse models, researchers unveiled that lack of TG2 in the TME prolonged survival in the metastatic model, but not the non-metastatic model. Loss of host TG2 decreased immunosuppressive TAMs while increasing T, natural killer, and B cells. Findings suggest that TG2 in the TME drives metastasis, possibly by modulating B cell activation and dampening humoral immunity.

The review by Liu et al. outlines the pivotal roles of innate immune cells, including macrophages, neutrophils, natural killer cells, dendritic cells, and myeloid-derived suppressor cells in either promoting or suppressing colorectal cancer progression through cytokine signaling, metabolic reprogramming, and interactions between tumor cells and immune cells. It emphasizes their contributions to inflammation, angiogenesis, immune evasion, and metastasis. The article highlights that emerging immunotherapies targeting these innate immune components show promise but remain limited, underscoring the need for deeper mechanistic understanding and improved combination strategies.

Macrophage migration inhibitory factor (MIF) is a key cytokine central to immune regulation, inflammation, and tumorigenesis. Initially known for inhibiting macrophage migration, MIF is now recognized as a potent driver in the progression of various solid tumors. The review by Youness et al. traces the history of MIF, highlighting its structure, receptor interactions, and signaling pathways. Crucially, it explores how MIF promotes tumor pathogenesis through enhanced proliferation, angiogenesis, immune evasion, and metastasis. Special focus is given to the influence of MIF on oncogenic pathways and its role in therapeutic resistance. Ultimately, the article discusses MIF's potential as a valuable diagnostic biomarker and therapeutic target in solid tumors.

Tumor-associated tertiary lymphoid structures (TLSs) are immune-responsive aggregates linked to better prognosis in various cancers. The study by Xi et al. examined TLSs in 171 patients with extramammary Paget's disease (EMPD), revealing their presence in 57% of the cases. Mature TLSs were associated with tumor invasion and recurrence, suggesting their role in immune response. The study findings indicate that mature TLSs could serve as a prognostic biomarker and potential therapeutic target for invasive EMPD, highlighting the need for further research on their clinical applications.

The study by Xu et al. on esophageal squamous cell carcinoma (ESCC) examined the impact of HLA-E expression and natural killer cell proportion on patients' prognosis. An immunosuppression score (ISS) was developed, revealing that low ISS had significantly better survival outcomes. Further, low HLA-E expression and natural killer cell levels correlated with higher recurrence and mortality. Findings suggested ESCC to be comprised of distinct biological subtypes, emphasizing the need for personalized treatment strategies. These insights could refine cancer prognosis and guide more effective therapeutic approaches.

Immunotherapy has revolutionized cancer treatment, yet its efficacy remains limited across various cancers. The review by Murakami and Ganguly discussed the emergence of poliovirus receptor-related 2 (PVRL2) and poliovirus receptor (PVR), members of the Nectin family, as key immune checkpoint factors suppressing anti-tumor immunity. As they are highly expressed in gynecological as well as other solid and hematologic tumors, they interact with PVR related immunoglobulin domain (PVRIG) and T cell immunoreceptor with Ig and ITIM domains (TIGIT) on T and natural killer cells. Targeting these pathways offers a promising therapeutic strategy. Also, the role of the complex DNAM-1/CD226 axis was explored in cancer immunotherapy, highlighting potential advancements in treatment.

In their review, Wang et al. describes how natural killer cells become functionally impaired in myelodysplastic syndromes due to inhibitory influences from the TME, stromal components, metabolic conditions, cancer stem cells, and gene mutations. These factors reduce natural killer cell cytotoxicity, activation receptor expression, and metabolic capacity. The article also summarizes therapeutic advances, including chimeric antigen receptor–natural killer cell therapy, allogeneic and autologous natural killer cell transfer, cytokine-based activation, and immune checkpoint inhibition, emphasizing their potential to restore anti-tumor immunity in myelodysplastic syndromes.

The article by Donnenberg et al. shows that epithelial cancers metastatic to the pleura share a common secretomic profile dominated by cytokine interleukin-6 and its soluble receptor. It emphasizes that the pleural environment, rather than tumor genetics, drives aggressive disease. Furthermore, it demonstrates that the pleural cavity functions as a bioreactor, generating a cytokine milieu that promotes epithelial to mesenchymal transition and suppresses immunity. The interleukin-6 signaling axis acts as a unifying pathogenic mechanism and a promising therapeutic target across pleural metastases.

Immunotherapy for hematological malignancies faces limitations due to drug resistance, necessitating the discovery of novel targets. The article by Yang et al. highlights N6-methyladenosine (m6A), the most common RNA modification, as a pivotal factor in these cancers. Reportedly, m6A modifications influence the immune microenvironment, promoting immune evasion and compromising anti-tumor responses. This article comprehensively summarizes the roles of m6A modifications in various hematological cancers, focusing on their impact on immunity. It also reviews the progress in developing m6A-targeted drugs, offering new therapeutic insights.

The study by Zhang et al. assessed CAR-T-cell therapy for Multiple Myeloma (MM) using meta-analysis and Mendelian Randomization (MR). The meta-analysis of 34 articles showed an 82.2% overall response rate (ORR), with a safe profile (6.3% severe cytokine release syndrome, 0.9% neurotoxicity). BCMA, CD38, and GPRC5D targets yielded the best responses. MR analysis identified seven immune cell types associated with an increased MM risk and eight with a decreased risk. The study also linked risk genes like VDR and VHL to MM, supporting CAR-T efficacy and deepening the understanding of the underlying pathophysiology of MM.

Prolyl 3-hydroxylases (P3H) are vital for collagen synthesis, but their roles in cancer and the TME are under investigation. The study by Wang and Wang analyzed P3H family gene expression and clinical data from GTEx and TCGA to assess their prognostic significance. The study findings revealed that P3H proteins play diverse roles in cancer, with most P3H family genes, notably P3H1 and P3H4, acting as risk factors. The calculated P3H score correlated with immune infiltration and drug resistance. High expression of P3H2, P3H3, and CRTAP was linked to increased resistance to anti-tumor drugs. The P3H score is concluded to be a potential biomarker to guide precision medicine strategies.

In conclusion, this Research Topic provided a thorough overview of the multifaceted role of the TME in regulating anti-tumor immunity, highlighting novel therapeutic targets and mechanistic insights across a range of malignancies. The key takeaway is that resistance to cancer immunotherapy involves a dynamic interplay, not just of tumor cells, but also stromal components like CAFs, diverse immune subsets like TAMs and Tregs, complex signaling molecules like IL-6, MIF, and chemokines, and key epigenetic factors like m6A modifications. The findings suggest promising therapeutic avenues, such as targeting CAFs or utilizing novel agents like Sudocetaxel Zendusortide. Moving forward, sequential research must focus on the clinical translation of multi-targeting strategies-such as combining therapies that address stromal and immune suppression- validating dynamic predictive biomarkers like the ISS or P3H score to personalize treatment, and fully dissecting the crosstalk between metabolic, epigenetic, and immune regulation within the TME to identify master regulators for next-generation immunotherapies.

